# Antibiotic resistance among invasive *Neisseria meningitidis* isolates in England, Wales and Northern Ireland (2010/11 to 2018/19)

**DOI:** 10.1371/journal.pone.0260677

**Published:** 2021-11-29

**Authors:** Laura Willerton, Jay Lucidarme, Andrew Walker, Aiswarya Lekshmi, Stephen A. Clark, Lloyd Walsh, Xilian Bai, Lisa Lee-Jones, Ray Borrow

**Affiliations:** 1 Meningococcal Reference Unit, Public Health England, Manchester Royal Infirmary, Manchester, United Kingdom; 2 Life Sciences Department, Manchester Metropolitan University, Manchester, United Kingdom; Universidad Nacional de la Plata, ARGENTINA

## Abstract

Invasive meningococcal disease (IMD), caused by *Neisseria meningitidis*, can have a fatality rate as high as 10%, even with appropriate treatment. In the UK, penicillin is administered to patients in primary care whilst third generation cephalosporins, cefotaxime and ceftriaxone, are administered in secondary care. The first-choice antibiotic for chemoprophylaxis of close contacts is ciprofloxacin, followed by rifampicin. Immunocompromised individuals are often recommended antibiotic chemoprophylaxis and vaccination due to a greater risk of IMD. Resistance to antibiotics among meningococci is relatively rare, however reduced susceptibility and resistance to penicillin are increasing globally. Resistance to third generation cephalosporins is seldom reported, however reduced susceptibility to both cefotaxime and ceftriaxone has been observed. Rifampicin resistance has been reported among meningococci, mainly following prophylaxis, and ciprofloxacin resistance, whilst uncommon, has also been reported across the globe. The Public Health England Meningococcal Reference Unit receives and characterises the majority of isolates from IMD cases in England, Wales and Northern Ireland. This study assessed the distribution of antibiotic resistance to penicillin, rifampicin, ciprofloxacin and cefotaxime among IMD isolates received at the MRU from 2010/11 to 2018/19 (n = 4,122). Out of the 4,122 IMD isolates, 113 were penicillin-resistant, five were ciprofloxacin-resistant, two were rifampicin-resistant, and one was cefotaxime-resistant. Penicillin resistance was due to altered *penA* alleles whilst rifampicin and ciprofloxacin resistance was due to altered *rpoB* and *gyrA* alleles, respectively. Cefotaxime resistance was observed in one isolate which had an altered *penA* allele containing additional mutations to those harboured by the penicillin-resistant isolates. This study identified several isolates with resistance to antibiotics used for current treatment and prophylaxis of IMD and highlights the need for continued surveillance of resistance among meningococci to ensure continued effective use.

## Introduction

Invasive meningococcal disease (IMD), including meningitis and/or septicaemia, is a severe, life-threatening illness caused by the bacterium *Neisseria meningitidis* (the meningococcus). Six meningococcal serogroups (A, B, C, W, X and Y) are responsible for the majority of IMD cases worldwide [1–3]. Meningococci can be further differentiated into sequence types (STs), with related STs grouped into lineages termed clonal complexes (CCs) [4]. Serogroup B meningococcal disease is the most prevalent in the UK and is the most diverse in terms of clonal complex distribution [5].

IMD is fatal in 5–10% of cases in developed countries, despite prompt commencement of antibiotic treatment [6]. In the UK, patients in primary care with suspected meningococcal disease are administered penicillin, whilst those in secondary care are administered third generation cephalosporins (3GCs), ceftriaxone or cefotaxime [7]. Ciprofloxacin is the chemoprophylactic agent of choice for close contacts of cases and rifampicin is a suitable alternative [8]. Chemoprophylaxis is recommended for close contact of IMD cases and for individuals with deficiencies of the terminal complement pathway, where the risk for IMD development is up to 10,000 fold higher than in the general population [9].

*N*. *meningitidis* has remained largely susceptible to the antibiotics used in the treatment and prevention of IMD. Resistance to ciprofloxacin, due to alterations in the *gyrA* gene (encoding DNA gyrase subunit A), is relatively uncommon, except for in China, where the prevalence of resistance among isolates, in particular of the ST-4821 CC, has led to the withdrawal of its use as a first line prophylactic agent [10, 11]. More recently, ciprofloxacin resistance has been reported in invasive ST-23 CC isolates in the USA [12] and in invasive ST-175 CC isolates in immunocompromised individuals in the UK and Europe [13]. Mutations in the *parC* gene (encoding DNA topoisomerase IV, subunit A), when accompanied by mutations in the *gyrA* gene, cause enhanced levels of ciprofloxacin resistance [14].

Primary cases of rifampicin-resistant meningococci due to alterations in the *rpoB* gene (encoding the beta subunit of RNA polymerase), is uncommon and not associated with particular lineages. It is usually observed following rifampicin prophylaxis [15–17].

Penicillin resistance among meningococci due to the production of beta lactamase is rare, however, has recently been identified among ST-23 CC isolates in the USA [12]. Penicillin resistance and reduced susceptibility due to amino acid substitutions (AASs) (F504L, A510V, I515V, H541N, and I566V) in *penA*, encoding penicillin binding protein 2 (PBP2), are more frequently observed worldwide [18–21], and several countries have recently reported an increase in penicillin resistant invasive serogroup W ST-11 CC isolates [22, 23].

Meningococcal resistance to 3GCs is rare. A report of cefotaxime and ceftriaxone resistance among serogroup A isolates in India [24] has been met with some scepticism [25]. Reduced susceptibility to cefotaxime has been observed among several invasive isolates in France [26]. The *penA* gene responsible, *penA327*, was identical to that causing reduced susceptibility to 3GCs in *N*. *gonorrhoea* [27]. The allele, containing only four out of the five aforementioned AASs, also causes penicillin resistance and reduced susceptibly among meningococci [20, 26]. Amino acid substitution A501P in *penA* has been shown to enhance levels of 3GC resistance among *N*. *gonorrhoea*, and there are some concerns regarding the potential selection of this same mutation among meningococci [28]. Surveillance of antibiotic resistance among this species therefore remains of paramount importance.

The Public Health England (PHE) Meningococcal Reference Unit (MRU) offers a complimentary national reference service for confirmation and characterisation of meningococcal isolates in England, Wales and Northern Ireland (E, W and NI). The aim of this study was to characterise isolates from culture-confirmed cases of IMD received at the MRU from July 2010 to June 2019 inclusive (epidemiological years 2010/11 to 2018/19), and to determine the distribution of antibiotic susceptibility.

## Materials and methods

The study included 4,122 IMD isolates received by the MRU from July 2010 to June 2019, inclusive.

### Serogrouping

Serogrouping was performed using the dot-blot ELISA as previously described [29].

### Antibiotic susceptibility testing

Antibiotic susceptibility testing for penicillin, cefotaxime, rifampicin and ciprofloxacin was performed using the Etest (bioMerieux UK Limited, Basingstoke, UK) method on either Mueller Hinton agar plates supplemented with 5% horse blood and 20 mg/L β-nicotinamide adenine dinucleotide (Oxoid Limited, Basingstoke, UK) (isolates received on or after 9^th^ May 2019) or Iso-sensitest blood agar supplemented with 20 mg/L NAD (isolates received prior to 9^th^ May 2019).

MIC values were interpreted according to the European Committee on Antimicrobial Susceptibly Testing (EUCAST; v11.0; 2021-01-01).

Isolates with penicillin MICs ≤0.06 mg/L were categorised as ‘susceptible, standard exposure (S)’ (where there is a high likelihood of therapeutic success using a standard dosing regimen of the agent) and isolates with penicillin MICs >0.25 mg/L were categorised as ‘resistant (R)’ (where there is a high likelihood of therapeutic failure even when there is increased exposure). Isolates with intermediate penicillin MICs were categorised as ‘susceptible, increased exposure (I)’ (where there is a high likelihood of therapeutic success because exposure to the agent is increased by adjusting the dosing regimen or by its concentration at the site of infection) [30].

### Beta-lactamase testing

Beta-lactamase testing was performed using Interlactam strips (MAST, UK) following the manufacturer’s instructions.

### Genotypic analyses

Draft genomes were available for all isolates on the Meningitis Research Foundation Meningococcus Genome Library (MGL; https://pubmlst.org/bigsdb?db=pubmlst_neisseria_mrfgenomes; accessed 29^th^ June 2020).

Genotypic data were obtained using the BLAST and/or Export Dataset tools [31, 32].

### *PenA* sequencing

Where necessary, a 402 bp fragment (*penA*) of the NEIS1753 (PBP2) gene (pubmlst.org) was characterised by PCR and Sanger sequencing, as previously described using primers penA1F and penA1R [33].

## Results

### Serogroup and clonal complex distribution

Among all isolates, 53% (n = 2,178) were serogroup B, 24% (n = 1,004) were serogroup W, 16% were serogroup Y (n = 651) and 6% were serogroup C (n = 237). The remaining isolates were NG (n = 34), serogroup W/Y (n = 11), serogroup E (n = 4), serogroup X (n = 2) and serogroup A (n = 1). Most of the isolates (n = 3,585; 87%) belonged to eight main clonal complexes (ST-11 CC, n = 1,138; ST-41/44 CC, n = 748; ST-23 CC, n = 585; ST-269 CC, n = 520; ST-213 CC, n = 245; ST-32 CC, n = 191; ST-22 CC, n = 80 and ST-461 CC, n = 78). The remaining isolates belonged to less prevalent CCs (n = 298) or were unassigned to a CC (n = 239).

### Distribution of antibiotic susceptibility

More than half of the isolates (63%; n = 2,591) were penicillin-susceptible, standard exposure (PenS; MICs 0.004–0.064 mg/L), 34% (n = 1,418) were penicillin-susceptible, increased exposure (PenI; MICs 0.094–0.25 mg/L) and 3% (n = 113) were penicillin-resistant (PenR; MICs 0.38–0.75 mg/L) (Table 1). The MIC_50_ for penicillin was 0.064 mg/L and the MIC_90_ was 0.19 mg/L. The highest proportion of PenR isolates were received in 2018/19 (7%).

**Table 1:** Number of isolates from culture-confirmed IMD cases by epidemiological year in England Wales and Northern Ireland, from 2010/11 to 2018/19.

	Number of isolates by epidemiological year (%)									
Susceptibility category (MIC) by antibiotic (mg/L)	2010/11 (n = 500)	2011/12 (n = 400)	2012/13 (n = 450)	2013/14 (n = 403)	2014/15 (n = 505)	2015/16 (n = 521)	2016/17 (n = 501)	2017/18 (n = 488)	2018/19 (n = 354)	Total (n = 4,122)
**Penicillin**										
PenS ≤0.06 mg/L	336 (67.2)	277 (69.3)	277 (61.6)	247 (61.3)	349 (69.1)	272 (52.2)	335 (66.9)	313 (64.1)	185 (52.3)	2,591 (62.9)
PenI 0.094–0.25 mg/L	157 (31.4)	123 (30.8)	164 (36.4)	148 (36.7)	150 (29.7)	223 (42.8)	150 (29.9)	159 (32.6)	144 (40.7)	1,418 (34.4)
PenR >0.25 mg/L	7 (1.4)	0 (0.0)	9 (2.0)	8 (2.0)	6 (1.2)	26 (5.0)	16 (3.2)	16 (3.3)	25 (7.1)	113 (2.7)
**Cefotaxime**										
Susceptible ≤0.125 mg/L	499	400	450	403	505	521	501	488	354	4,121
Resistant >0.125 mg/L	1	0	0	0	0	0	0	0	0	1
**Ciprofloxacin**										
Susceptible ≤0.03 mg/L	499	400	450	403	503	521	501	488	352	4,117
Resistant >0.03 mg/L	1	0	0	0	2	0	0	0	2	5
**Rifampicin**										
Susceptible ≤0.25 mg/L	500	400	449	403	505	520	501	488	354	4,120
Resistant >0.25 mg/L	0	0	1	0	0	1	0	0	0	2

PenS = Penicillin susceptible, standard exposure; PenI = Penicillin-susceptible, increased exposure; PenR = Penicillin-resistant according to EUCAST guidelines. The annual percentage of PenS, I and R isolates is also displayed.

One isolate was cefotaxime-resistant (MIC = 0.25 mg/L) and the remaining isolates were cefotaxime-susceptible (n = 4,121; MICs = <0.002–0.125 mg/L). The MIC_50_ for cefotaxime was 0.004 mg/L and the MIC_90_ was 0.008 mg/L.

Five isolates were ciprofloxacin-resistant (MICs = 0.06–0.5 mg/L), and 4,117 isolates were ciprofloxacin-sensitive (MICs = <0.002–0.03 mg/L). The MIC_50_ for ciprofloxacin was 0.004 mg/L and the MIC_90_ was 0.008 mg/L.

Two isolates were rifampicin-resistant (MICs = 0.5 and >32 mg/L) and 4,120 isolates were rifampicin-sensitive (MICs = <0.002–0.25 mg/L). The MIC_50_ for rifampicin was 0.008 mg/L and the MIC_90_ was 0.023 mg/L.

Only one isolate was resistant to more than one antibiotic (penicillin and cefotaxime; 0.5 mg/L and 0.25 mg/L, respectively; PubMLST ID 20267). Details of all isolates that were resistant to at least one antibiotic are listed in S1 Table.

### Penicillin susceptibility

**Fig 1:**
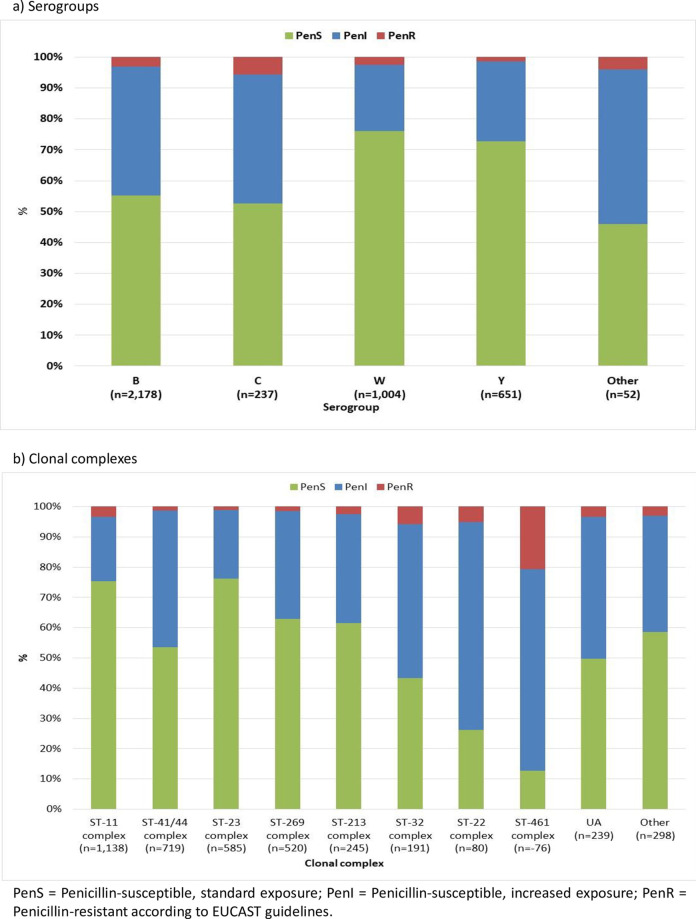
Penicillin susceptibility among serogroups and clonal complexes of IMD isolates in England, Wales and Northern Ireland, 2010/11 to 2018/19.

Fifty-five percent of serogroup B isolates were penicillin-susceptible (Fig 1). The proportion of penicillin-susceptible serogroup C, W and Y isolates were 53%, 76% and 73%, respectively. The proportion of serogroup B PenS isolates decreased from 67% in 2010/11 to 38% in 2018/19 (S1 Fig). There was an increase in the proportion of serogroup W PenR isolates over the last four years, with the highest proportion observed in 2018/19 (8%; S1 Fig).

The most prevalent clonal complex (all serogroups) was ST-11 CC followed by ST-41/44 CC where 75% and 56% of isolates were penicillin-susceptible, respectively (Fig 1). The clonal complex with the lowest proportion of PenS isolates was ST-461 CC (13%), which was also associated with the highest proportion of PenR isolates (21%).

**Fig 2:**
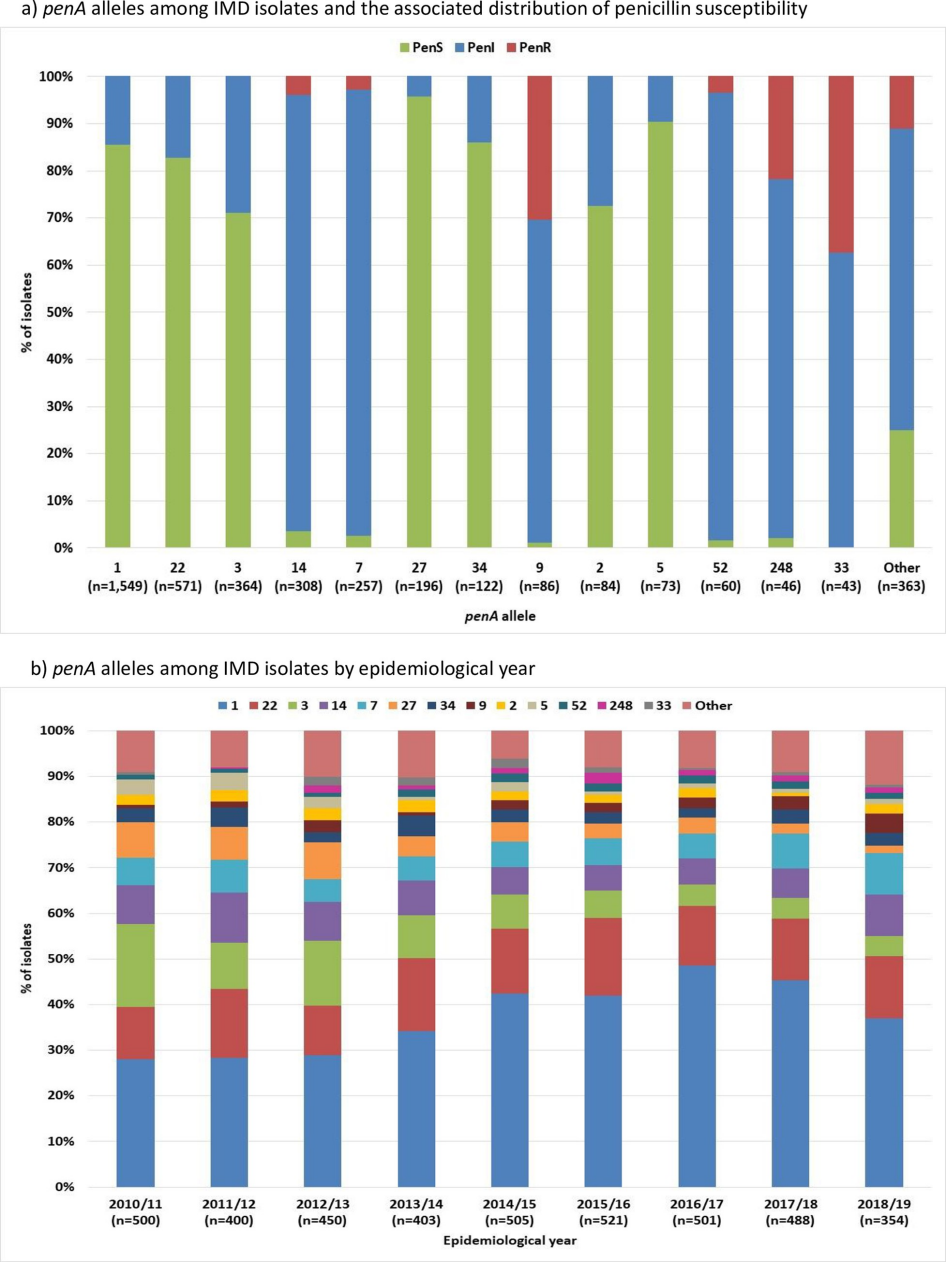
*penA* alleles among IMD isolates in England, Wales and Northern Ireland 2010/11 to 2018/19.

In total, 133 *penA* alleles were identified. *PenA1* was most prevalent (n = 1,549, 38%), followed by *penA22* (n = 571, 14%), for which 86% and 83% of isolates were penicillin-susceptible, respectively (Fig 2). *PenA33* (n = 43, 1%) was associated with the highest proportion of PenR isolates at 37%. The proportion of isolates with *penA3* and *penA27* decreased over time and, over the last four years, the proportion of isolates with *penA9* increased (Fig 2).

**Fig 3:**
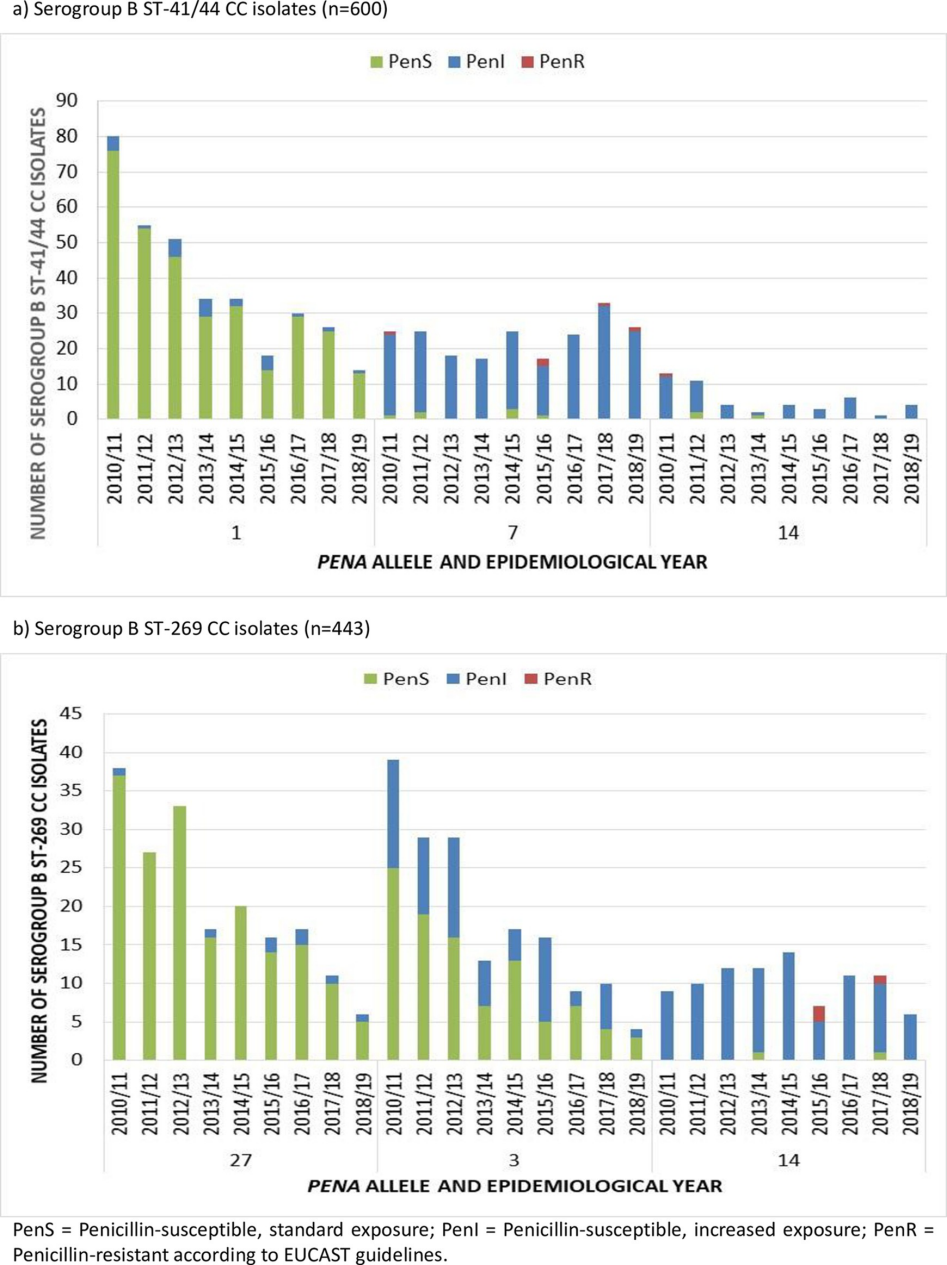
Prevalent *penA* alleles among serogroup B IMD isolates belonging to ST-41/44 and ST-269 clonal complex in England, Wales and Northern Ireland 2010/11 to 2018/19.

The increase in PenR serogroup W isolates over the last four years (S1 Fig) was due to a rise in the number of serogroup W ST-11 CC isolates harbouring *penA9*, as reported previously [22]. The decrease of serogroup B PenS isolates overtime (S1 Fig) was largely due to a decrease in serogroup B PenS ST-41/44 CC isolates with *penA1* and ST-269 CC isolates with *penA3* and *penA27* (Fig 3).

Thirty-one *penA* alleles were identified among the 113 PenR isolates (Table 2). All but three of these had the five AASs associated with penicillin resistance and reduced susceptibility (F504L, A510V, I515V, H541N and I566V). *PenA171*, found in one PenR isolate, had none of these mutations. *PenA209*, found in one PenR isolate had three out of the five AASs (F504L, A510V and I515V) and *PenA327*, found among PenR (n = 1) PenI (n = 3) and PenS (n = 1) isolates, had four out of the five AASs (F504L, A510V, I515V, H541N). All isolates with *penA327* (n = 5) also displayed reduced susceptibility to cefotaxime (0.047–0.125 mg/L).

**Table 2:** *PenA* alleles among PenR and PenI IMD isolates in England, Wales and Northern Ireland from 2010/11 to 2018/19.

*penA* allele	Total number of isolates	Number of PenS isolates	Number of PenI isolates	Number of PenR isolates	MIC value or range (mg/L)	MIC50	MIC90	Number of penicillin resistance associated AASs
1	1549	1327	222	0	0.004–0.25	0.047	0.094	0
2	84	61	23	0	0.016–0.25	0.064	0.125	0
3	364	259	105	0	0.047–0.25	0.064	0.094	0
4	13	11	2	0	0.016–0.094	0.047	0.094	0
5	73	66	7	0	0.012–0.19	0.047	0.064	0
7	257	7	243	7	0.064–0.38	0.19	0.25	5
9	86	1	59	26	0.047–0.75	0.25	0.38	5
10	5	0	2	3	0.25–0.38			5
11	1	0	0	1	0.5			5
12	16	0	14	2	0.125–0.5	0.19	0.38	5
13	7	0	5	2	0.125–0.38			5
14	308	11	285	12	0.047–0.5	0.19	0.25	5
16	9	8	1	0	0.023–0.094			0
19	22	1	18	3	0.047–0.38	0.19	0.38	5
20	6	0	5	1	0.125–0.5			5
21	3	0	1	2	0.19–0.38			5
22	571	473	98	0	0.012–0.19	0.064	0.094	0
25	3	0	2	1	0.125–0.38			5
27	196	188	8	0	0.008–0.125	0.047	0.064	0
33	43	0	27	16	0.094–0.75	0.25	0.5	5
34	122	105	17	0	0.012–0.19	0.047	0.094	0
42	11	0	10	1	0.125–0.38	0.19	0.25	5
52	60	1	57	2	0.064–0.38	0.19	0.25	5
54	1	0	0	1	0.38			5
62	5	3	2	0	0.032–0.094			0
66	5	0	5	0	0.094–0.25			5
83	4	2	2	0	0.047–0.125			0
90	5	0	5	0	0.125–0.19			5
91	4	0	4	0	0.125–0.19			5
110	2	0	1	1	0.125–0.38			5
119	4	1	3	0	0.032–0.125			0
157	15	14	1	0	0.032–0.094	0.047	0.064	0
171	1	0	0	1	0.38			0
179	1	0	0	1	0.38			5
209	1	0	0	1	0.5			3
238	5	0	5	0	0.094–0.19			5
248	46	1	35	10	0.047–0.38	0.25	0.38	5
295	6	0	3	3	0.25–0.5			5
327	5	1	3	1	0.047–0.38			4
331	1	0	0	1	0.5			5
342	4	2	2	0	0.047–0.094			0
348	16	2	11	3	0.047–0.38	0.125	0.38	5
371	5	0	4	1	0.125–0.38			5
386	10	1	9	0	0.047–0.125	0.125	0.125	5
414	8	0	7	1	0.125–0.38			5
419	1	0	0	1	0.5			5
420	8	0	8	0	0.125–0.25			5
435	1	0	0	1	0.38			5
540	6	0	1	5	0.25–0.38			5
593	1	0	0	1	0.38			5
921	1	0	0	1	0.38			5

Alleles among n = 3,981 IMD isolates. Alleles among PenI IMD isolates represented by 4+ isolates only. PenS = Penicillin-susceptible, standard exposure; PenI = Penicillin-susceptible, increased exposure; PenR = Penicillin-resistant according to EUCAST guidelines. MIC_50_ and MIC_90_ calculated for ≥10 isolates. AASs = amino acid substitutions.

### Cefotaxime susceptibility

Seven isolates had cefotaxime MICs ≥ 0.047 mg/L (0.2%), including one resistant isolate (MIC = 0.25 mg/L). These isolates harboured either *penA327* (n = 5; MIC = 0.047–0.125 mg/L), *penA11* (n = 1; MIC = 0.047 mg/L) or *penA419* (n = 1; MIC = 0.25 mg/L). Bar one, these isolates were also PenR (0.38–0.5 mg/L) or PenI (0.125–0.25 mg/L).

Allele *penA419*, which was also associated with penicillin resistance, was not found in any other isolates on PubMLST (accessed 10^th^ June 2021). In addition to the five AASs associated with penicillin resistance, the allele had substitutions A501T and D511V that were not found among *penA* alleles harboured by cefotaxime-susceptible PenR isolates received at the MRU.

Isolates with *penA* alleles harbouring AASs at positions 501 and 511 on PubMLST (n = 56; Table 3) included *N*. *meningitidis* (n = 50), *N*. *lactamica* (n = 5) and *N*. *polysaccharea* (n = 1). All *penA* alleles amongst these isolates had A501T (n = 17) or A501V (n = 39). Six isolates that had a *penA* allele with A501T also had an additional AAS at position 511; D511A (*penA419*; n = 1), D511G (*penA910*; n = 2, *penA836*; n = 1) or D511V (*penA670*; n = 1, *penA805*; n = 1). Where known, cefotaxime MICs of isolates with *penA* alleles with A501V only were 0.003–0.012 mg/L (n = 14). Isolates with *penA* alleles with A501T only had MICs of 0.016–0.125 mg/L (n = 6). Isolates with both A501T and D511 mutations in the *penA* alleles (*penA419*, *penA910*) had MICs of 0.125–0.5 mg/L (n = 3).

**Table 3:** Isolates with *penA* alleles harbouring AASs at positions 501 and 511 on PubMLST.

PubMLST ID	Country	Year	Site	Species	Cefotaxime MIC (mg/L)	Penicillin MIC (mg/L)	*penA* allele	A501 mutation	D511 mutation	Other 5 penicillin resistance-associated mutations*?
20267	England	2011	Blood	*Neisseria menigitidis*	0.25	0.5	419	A501T	D511A	Yes
72882	Greece	2020	NK	*Neisseria menigitidis*	0.125	0.5	910	A501T	D511G	Yes
72883	Greece	2020	NK	*Neisseria menigitidis*	0.19	0.5	910	A501T	D511G	Yes
92874	China	2015	Throat	*Neisseria lactamica*	NK	NK	836	A501T	D511G	Yes
38947	Italy	2013	Throat	*Neisseria menigitidis*	NK	NK	670	A501T	D511V	Yes
41652	Italy	2015	NK	*Neisseria polysaccharea*	NK	NK	805	A501T	D511V	Yes
93629	Germany	2016	Blood	*Neisseria menigitidis*	0.047	0.25	909	A501T	None	Yes
93630	Germany	2016	Blood	*Neisseria menigitidis*	0.047	0.5	909	A501T	None	Yes
17230	Niger	1961	CSF	*Neisseria menigitidis*	NK	0.016	84	A501T	None	No
15965	France	2010	CSF	*Neisseria menigitidis*	0.047	0.125	341	A501T	None	Yes
16970	France	2005	CSF	*Neisseria menigitidis*	0.125	0.38	61	A501T	None	Yes
45332	France	2016	NK	*Neisseria menigitidis*	0.016	0.38	734	A501T	None	Yes
28074	England	2013	Blood	*Neisseria menigitidis*	0.004	0.012	24	A501V	None	No
35318	Scotland	2011	CSF	*Neisseria menigitidis*	0.004	0.015	24	A501V	None	No
35288	Scotland	2010	Blood	*Neisseria menigitidis*	0.004	0.023	411	A501V	None	No
35437	England	2013	Blood	*Neisseria menigitidis*	0.008	0.023	311	A501V	None	No
19771	France	2011	Blood	*Neisseria menigitidis*	0.06	0.023	311	A501V	None	No
20303	England	2011	Blood	*Neisseria menigitidis*	0.003	0.032	311	A501V	None	No
60835	England	2017	Blood	*Neisseria menigitidis*	0.006	0.032	161	A501V	None	No
19794	France	2011	Blood	*Neisseria menigitidis*	0.008	0.032	311	A501V	None	No
38060	England	2015	Blood	*Neisseria menigitidis*	0.008	0.032	311	A501V	None	No
53225	England	2017	Blood	*Neisseria menigitidis*	0.008	0.032	161	A501V	None	No
20779	UK	NK	Blood	*Neisseria menigitidis*	0.006	0.047	24	A501V	None	No
44717	Northern Ireland	2016	Blood	*Neisseria menigitidis*	0.006	0.047	671	A501V	None	No
35716	England	2014	Blood	*Neisseria menigitidis*	0.012	0.047	24	A501V	None	No
20401	England	2011	Blood	*Neisseria menigitidis*	0.008	0.064	24	A501V	None	No
16899	France	2004	CSF	*Neisseria menigitidis*	NK	0.094	24	A501V	None	No
63353	Brazil	2016	CSF	*Neisseria menigitidis*	NK	NK	61	A501T	None	Yes
43592	England	2014	Throat	*Neisseria lactamica*	NK	NK	605	A501T	None	Yes
43618	England	2015	Throat	*Neisseria lactamica*	NK	NK	611	A501T	None	Yes
44105	England	2015	Throat	*Neisseria lactamica*	NK	NK	611	A501T	None	Yes
52723	England	2015	Throat	*Neisseria lactamica*	NK	NK	611	A501T	None	Yes
4193	England	1999	Throat	*Neisseria menigitidis*	NK	NK	311	A501V	None	No
7302	Poland	2001	CSF	*Neisseria menigitidis*	NK	NK	161	A501V	None	No
19907	Sweden	2010	Blood	*Neisseria menigitidis*	NK	NK	311	A501V	None	No
20803	UK	NK	Throat	*Neisseria menigitidis*	NK	NK	503	A501V	None	No
26051	Ivory Coast	1998	NK	*Neisseria menigitidis*	NK	NK	24	A501V	None	No
27068	Czech Republic	2013	Blood	*Neisseria menigitidis*	NK	NK	411	A501V	None	No
29312	South Africa	2006	NK	*Neisseria menigitidis*	NK	NK	519	A501V	None	No
30357	Australia	2005	NK	*Neisseria menigitidis*	NK	NK	311	A501V	None	No
30691	Ireland	2014	Blood	*Neisseria menigitidis*	NK	NK	311	A501V	None	No
31213	England	2013	Blood	*Neisseria menigitidis*	NK	NK	161	A501V	None	No
38224	Finland	2015	NK	*Neisseria menigitidis*	NK	NK	660	A501V	None	No
39150	Finland	2013	NK	*Neisseria menigitidis*	NK	NK	311	A501V	None	No
40185	France	2013	NK	*Neisseria menigitidis*	NK	NK	311	A501V	None	No
42510	England	2016	Blood	*Neisseria menigitidis*	NK	NK	24	A501V	None	No
46605	Wales	2015	Throat	*Neisseria menigitidis*	NK	NK	740	A501V	None	Yes
47204	Sweden	NK	NK	*Neisseria menigitidis*	NK	NK	311	A501V	None	No
49424	Wales	2015	Throat	*Neisseria menigitidis*	NK	NK	740	A501V	None	No
49490	Wales	2015	Throat	*Neisseria menigitidis*	NK	NK	740	A501V	None	No
52734	Finland	2017	NK	*Neisseria menigitidis*	NK	NK	311	A501V	None	No
56647	China	2012	Blood	*Neisseria menigitidis*	NK	NK	311	A501V	None	No
56697	Canada	2004	NK	*Neisseria menigitidis*	NK	NK	24	A501V	None	No
57042	England	1999	Throat	*Neisseria menigitidis*	NK	NK	671	A501V	None	No
82544	Finland	NK	NK	*Neisseria menigitidis*	NK	NK	311	A501V	None	No
88917	Japan	2007	Sputum	*Neisseria menigitidis*	NK	NK	311	A501V	None	No

NK = Not known.

* F504L, A510V, I515V, H541N, I566V.

### Ciprofloxacin susceptibility

Five isolates were resistant to ciprofloxacin (0.06–0.5 mg/L; S1 Table) with five different *gyrA* alleles; *gyrA6* (MIC = 0.5 mg/L), *gyrA8* (MIC = 0.18 mg/L), *gyrA10* (MIC = 0.094 mg/L), *gyrA146* (MIC = 0.19 mg/L) and *gyrA296* (MIC = 0.06 mg/L). These alleles were not harboured by any other IMD isolate received at the MRU from 2010/11–2018/19.

The *gyrA* alleles among the ciprofloxacin resistant isolates had AASs D95N (n = 2; MICs = 0.06–0.094 mg/L) or T91I (n = 3; MICs = 0.18–0.5 mg/L). The ciprofloxacin-resistant isolate with the highest ciprofloxacin MIC value (0.5 mg/L) also harboured a mutation in *parC* (D86N).

### Rifampicin susceptibility

Two isolates (ST-11 CC and ST-41/44 CC, respectively) were rifampicin resistant (MICs = 0.5 and >32 mg/L; S1 Table). These harboured *rpoB* alleles; *rpoB238* and *rpoB84*, respectively. *RpoB238* had AAS D542E and *RpoB84* had AAS H552N. There was no evidence that suggested either of the two isolates were from close contacts of index cases.

## Discussion

From 2010/11 to 2018/19, antibiotic resistance was uncommon among IMD isolates in E, W and NI. However, resistance to all four antibiotics (penicillin, rifampicin, cefotaxime and ciprofloxacin) was observed.

As has been reported in several other countries [20, 23, 34], the proportion of PenR isolates in E, W & NI has increased over time, thus, highlighting the need for surveillance of penicillin susceptibility in order to ensure its continued effective use as a first-line prophylactic agent.

The recent increase was largely due to PenR serogroup W ST-11 CC isolates that have emerged in a lineage that originated in South America in the early 2000s, and that has since spread to Europe, Australasia and North America [35–37]. Since 2016, Australia and the UK have both observed an increase in PenR isolates within the so-called original UK W:cc11 strain or the South American strain sublineage [22, 23]. Reports of penicillin-resistant meningococcal strains causing IMD in complement deficient individuals receiving chemoprophylaxis [38, 39] make this strain particular cause for concern.

Out of the 113 PenR isolates, three had a *penA* allele (*penA327*, *penA209* and *penA171*) that did not contain all five of the AASs commonly associated with penicillin resistance (F504L, A510V, I515V, H541N, I566V).

*PenA327*, (I566V absent), has been previously reported in penicillin-resistant meningococcal isolates and also those with reduced susceptibility to 3GCs [26]. All IMD isolates with *penA327* received at the MRU displayed reduced susceptibility to cefotaxime (MICs 0.047–0.125 mg/L).

*PenA209* had only three of the five AASs (F504L, A510V, I515V) alongside V447L and A516G. *PenA* alleles identified on PubMLST that contained F504L, A510V and I515V without V447L and A516 had lower penicillin MICs (0.047–0.094 mg/L) compared to those that also had V447L and A516 (0.12–0.5 mg/L). This may suggest that *penA* alleles harbouring the three AASs may not be adequate to confer penicillin resistance alone, however, do when in combination with V447 and A516. Future work is needed to confirm the role that these AASs have in conferring resistance to penicillin among meningococci.

*PenA171* contained none of the five AASs commonly associated with conferring reduced susceptibility and resistance to penicillin. It did contain P551S, a mutation which has been observed among gonococcal isolates resistant to penicillin [40], however, other meningococcal isolates with *penA171* on the PubMLST database were penicillin-susceptible, suggesting *penA171*, and the P551S AAS alone, are not responsible for causing penicillin resistance among meningococci. Further work is required to determine the cause of penicillin resistance in this isolate which was beta-lactamase negative, as reflected in the relatively low MIC (0.38 mg/L). Other mechanisms of penicillin resistance have been determined among *N*. *gonorrhoeae*, which include mutations in the transcriptional repressor of the efflux pump (mtrCDE) operon, which is encoded by the *mtrR* gene [41]. The operation of the mtrCDE efflux pump in meningococci appears to differ than in that of *N*. *gonorrhoeae* [42], with no evidence to suggest that mutations in the *mtrR* gene of meningococci can cause penicillin resistance.

Only seven isolates had cefotaxime MICs ≥ 0.047 mg/L, one of which was cefotaxime-resistant (MIC = 0.25 mg/L). Five of the seven isolates harboured *penA327*, which has previously been associated with reduced susceptibility to cefotaxime and penicillin in meningococci. Four of these were serogroup C isolates belonging to ST-11 CC and one was a serogroup B strain belonging to ST-41/44 CC. The allele has been previously identified among ST-11 CC isolates of serogroups B and C, suggesting successful clonal expansion of this particular strain [26]. The allele is identical to that of an allele identified among gonococcal isolates displaying resistance to ceftriaxone in this species [27].

Apart from *penA327*, no other *penA* allele has been associated with reduced susceptibility or resistance to 3GCs among meningococci. High levels of 3GC resistance due to A501P have been observed among gonococcal isolates [43]. Other AASs at this position close to the core of the active site motif of PBP2, A501T and A501V, have been described among gonococcal isolates displaying reduced susceptibility to cefotaxime [44–46]. It is thought that the substitution of methyl side chain alanine (A501) interferes with 3GC binding, or leads to structural modifications causing higher levels of 3GC resistance [43].

The cefotaxime-resistant meningococcal isolate identified in this study harboured *penA419* with two unique AASs, A501T and D511V, when compared to cefotaxime-susceptible penicillin-resistant isolates. Among other isolates on PubMLST with *penA* AASs at positions 501 and 511, isolates harbouring A501V had lower MICs than those with A501T. This suggests that AAS A501V does not confer reduced susceptibility to cefotaxime in meningococci, unlike in gonococci [45], whilst A501T may. When A501T was present in combination with an AAS at 511 (D511V or D511G), cefotaxime MICs among the meningococcal isolates were further enhanced. Amino acid substitutions at D511 are extremely rare. Further work is therefore required to confirm the role that this AAS plays in susceptibility to cefotaxime among meningococci. No meningococcal isolates with AAS A501P were identified, however, it has already been suggested that this mutation may eventually be selected in meningococci [28].

Five isolates were ciprofloxacin-resistant (MICs = 0.06–0.5 mg/L). Each of these harboured AASs in the *gyrA* gene (D95N or T91I) which have previously been associated with conferring ciprofloxacin resistance among meningococcal isolates worldwide [47–49]. The ciprofloxacin-resistant isolate with the highest MIC (MIC = 0.5 mg/L) also harboured an AAS in its *parC* allele (D86N). Mutations in *parC* among meningococcal isolates which also harbour mutations in *gyrA* have been shown to cause enhanced levels of resistance to ciprofloxacin (MICs of 0.5–1 mg/L).

The five ciprofloxacin-resistant isolates received at the MRU belonged to five different clonal complexes. In China, where ciprofloxacin resistance is prevalent, resistance is commonly seen among isolates belonging to ST-4821 CC, which has also been observed among ciprofloxacin-resistant isolates in other countries [10, 48, 50]. In the USA, a recent study identified ciprofloxacin resistance among several meningococcal isolates belonging to ST-23 CC [12]. Since the ciprofloxacin-resistant isolates in the present study do not seem to be prevalent among a certain clonal complex, it may suggest that these particular strains in the UK have not successfully expanded with the altered *gyrA* alleles and indicates that levels of ciprofloxacin resistance may continue to remain low within the population. Recently however, ciprofloxacin resistance has been observed among a particular strain of NG meningococci of the ST-175 CC in the UK and Europe, causing IMD in immunocompromised individuals. Occasionally, ciprofloxacin may be recommended to immunocompromised individuals or prescribed as a rescue therapy for when symptoms of IMD occur despite other prophylactic regimes [51–53] and so the presence of this non-groupable ST-175 CC strains in the UK remains a concern.

Only two isolates were resistant to rifampicin which is rare among meningococci considering its widespread use as a prophylactic agent. The majority of meningococcal rifampicin resistance is found among isolates from cases of close contacts following rifampicin prophylaxis [15, 17]. However, rifampicin resistance has also been observed among index cases [54]. There was no evidence to suggest that the two rifampicin-resistant isolates received at the MRU were from close contact cases.

One rifampicin-resistant isolate harboured *rpoB238* (MIC = 0.5 mg/L), which had AAS 542E. The other rifampicin-resistant isolate *rpoB84* (MIC = >32 mg/L), which had AAS H552N. Both AASs have been previously reported among rifampicin-resistant meningococcal isolates, with mutations at AA position 552 associated with higher levels of resistance than those with mutations at AA position 542 [55]. Studies have also identified the same AASs among multiple isolates with varying levels of rifampicin resistance [54, 56]. This, along with the observed difference in rifampicin MIC values displayed by the two resistant isolates in this study, may indicate that additional unknown mechanisms can cause increased levels of rifampicin resistance among meningococci.

This study identified several IMD isolates with resistance to antibiotics used for current treatment and prophylaxis of IMD. Future genetic engineering work is needed to confirm the role that certain *penA* mutations have in conferring resistance to penicillin and cefotaxime among meningococci. Further molecular work is also required to determine possible alternative penicillin resistance mechanisms of meningococci. This study also identified emerging threats and the progression of antibiotic resistance among strains over time. Consideration should be given before the mass use of antibiotics in single-dose prophylaxis strategies to control outbreaks of meningococcal disease, particularly where outbreaks frequently occur such as in the African meningitis belt [57]. Sustained surveillance of antibiotic resistance among the meningococcal population is essential to maintain successful treatment regimens for IMD and chemoprophylaxis regimes.

## Supporting information

S1 Fig Annual distribution of penicillin susceptibility among serogroup B, C, W and Y IMD isolates in England, Wales and Northern Ireland, 2010/11 to 2018/19. (TIF)

S1 Table Isolates from culture-confirmed cases of IMD in E, W and NI 2010/11-2018/19 resistant to at least one of the antibiotics tested. (XLSX)
