# Supplementary figures and images for: Antibiotic resistance among invasive Neisseria meningitidis isolates in England, Wales and Northern Ireland (2010/11 to 2018/19)
Source: PLoS One. 2021 Nov 29;16(11):e0260677. doi: 10.1371/journal.pone.0260677 (PMC8629238; doi:10.1371/journal.pone.0260677)

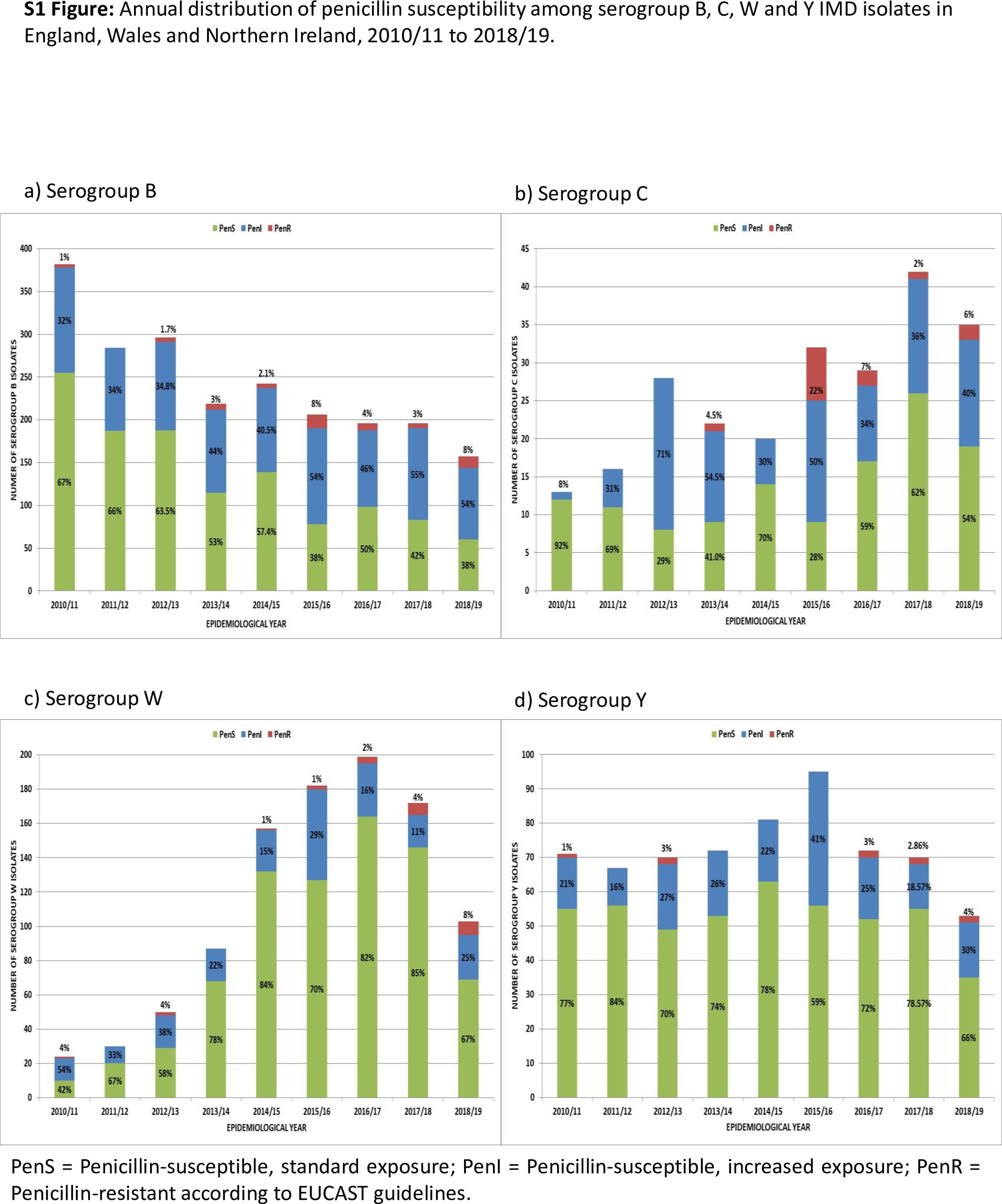

Supplement: S1 Fig — (TIF) [file pone.0260677.s001.tif]
